# Potential of Bacterial Isolates from a Stream in Manaus-Amazon to Bioremediate Chromium-Contaminated Environments

**DOI:** 10.1007/s11270-018-3903-1

**Published:** 2018-07-31

**Authors:** Ydrielly Veras Teles, Lorena Mota de Castro, Ézio Sargentini Junior, Aryana Pinheiro do Nascimento, Henrique Alves da Silva, Rebeca Silveira Costa, Rayane Delmontes do Nascimento Souza, Adolfo José da Mota, José Odair Pereira

**Affiliations:** 1 Universidade Federal do Amazonas, Av. General Rodrigo Octávio Jordão Ramos, 1200, Manaus, Amazonas Brazil; 2 Instituto Nacional de Pesquisas da Amazônia, Av. André Araújo, 2396, Manaus, Amazonas Brazil; 3 Universidade do Estado do Amazonas, Av. Djalma Batista, 2470, Manaus, Amazonas Brazil

**Keywords:** Cr(VI) degradation, Amazonian stream, *Vagococcus* sp., Industrial area

## Abstract

*Igarapé do Quarenta* (IgQ), a stream located in the Manaus-AM, BR, has directly experienced the impacts of urban expansion over the last five decades, which contributed for its contamination. As an affluent of *Rio Negro*, IgQ also affects the water quality of this important river that bathes Manaus. However, the stress caused by the prolonged exposition to chemical agents may have selected microorganisms that exhibit great bioremediation potential. In the present study, bacteria isolated from four distinct sites of the IgQ were identified, and their potential to degrade hexavalent chromium (Cr(VI)) was investigated using the s-diphenylcarbazide method. Among the investigated isolates, 14 exhibited resistance against Cr(VI) at a concentration of 300 mg/L and eight isolates reduced over 50% (53.5–97.4%) chromium ratios after 72 h of incubation. Those isolates were identified by gene sequencing and classified in 10 genera (*Acidovorax* sp., *Acinetobacter* sp., *Alicycliphilus* sp., *Bacillus* sp., *Comamonas* sp., *Enterobacter* sp., *Micrococcus* sp., *Proteus* sp., *Serratia* sp., and *Vagococcus* sp.). Under control conditions, the isolate of *Vagococcus* sp. genus, in only 24 h of incubation, reduced 96.8% of the rate of Cr(VI) added to the culture medium at the concentration of 10 mg/L. Obtained results indicate that the *Vagococcus* sp. exhibits a great potential to be used in the bioremediation of areas contaminated with chromium. The mechanisms of action of microorganisms should be investigated for more specific applications in the decontamination of effluents and direct use of its by-products to bioremediate polluted environments.

**Electronic supplementary material:**

The online version of this article (10.1007/s11270-018-3903-1) contains supplementary material, which is available to authorized users.

## Introduction

In the east side of Manaus, AM, BR, there is a watercourse known as Igarapé do Quarenta (IgQ), which runs to the south side of the city where it meets the Igarapé do Educandos and flows into Rio Negro which is the largest affluent of the Amazon River. Since the establishment of Manaus Industrial District in the 1960s, the IgQ has being polluted with lots of domestic rejects and industrial effluents (Pinto et al. 2009; Torrezani et al. 2016).

This environmental problem requires special attention particularly in relation to the discharge of industrial effluents containing heavy metals, as it happens with chromium (Cr). Chromium metal is found in nature in the form of trivalent chromium, Cr(III), and hexavalent chromium, Cr(VI). While Cr(III) is considered a more stable form of chromium, less toxic, and of low solubility, the Cr(VI) is highly soluble, carcinogenic, teratogenic, and mutagenic. Cr is able to penetrate the cells via phosphate and sulphate transporters (Puzon et al. 2002; Codd et al. 2003; Asmatullah et al. 1998). According to the International Agency for Research on Cancer (IARC 1990), the Cr(VI) is classified as carcinogenic to humans (group 1), though chromium metal and trivalent chromium compounds are not classifiable as human carcinogens (group 3).

In order to minimize the impacts caused by hazardous waste, the potential of microorganisms for the bioremediation of environments polluted with heavy metals has been extensively investigated. The Amazonian biodiversity certainly represents a significant source for the prospection of microorganisms effective in bioremediation processes (Pereira et al. 2017).

In general, endemic bacteria from environments contaminated with metals are adapted and able to metabolize such biohazard compounds, showing a variety of resistance strategies, tolerance, and decontamination capacity (Fuller et al. 2015; Thatoi et al. 2014). The combination of high tolerance and resistance with the ability to degrade Cr(VI) to Cr(III) is one of the most important parameters to be considered in studies for the screening of microorganisms with bioremediation potential (Dhal et al. 2010). The bacterial tolerance to chromium may involve various mechanisms of interaction between the microbiota and the metal, for example, binding of the metal to the cell wall, reduction of cell membrane permeability, active extrusion, absorption, and formation of complexes with chelating agents such as metallothioneins (De Filippis and Pallaghy 1994; Reed and Gadd 1990).

Our aims were the isolation and identification of Cr(VI)-resistant bacteria collected from the IgQ waters and the evaluation of their potential to bioremediate chromium-contaminated environments.

## Material and Methods

### Study Area and Sample Collection

**Fig. 1:**
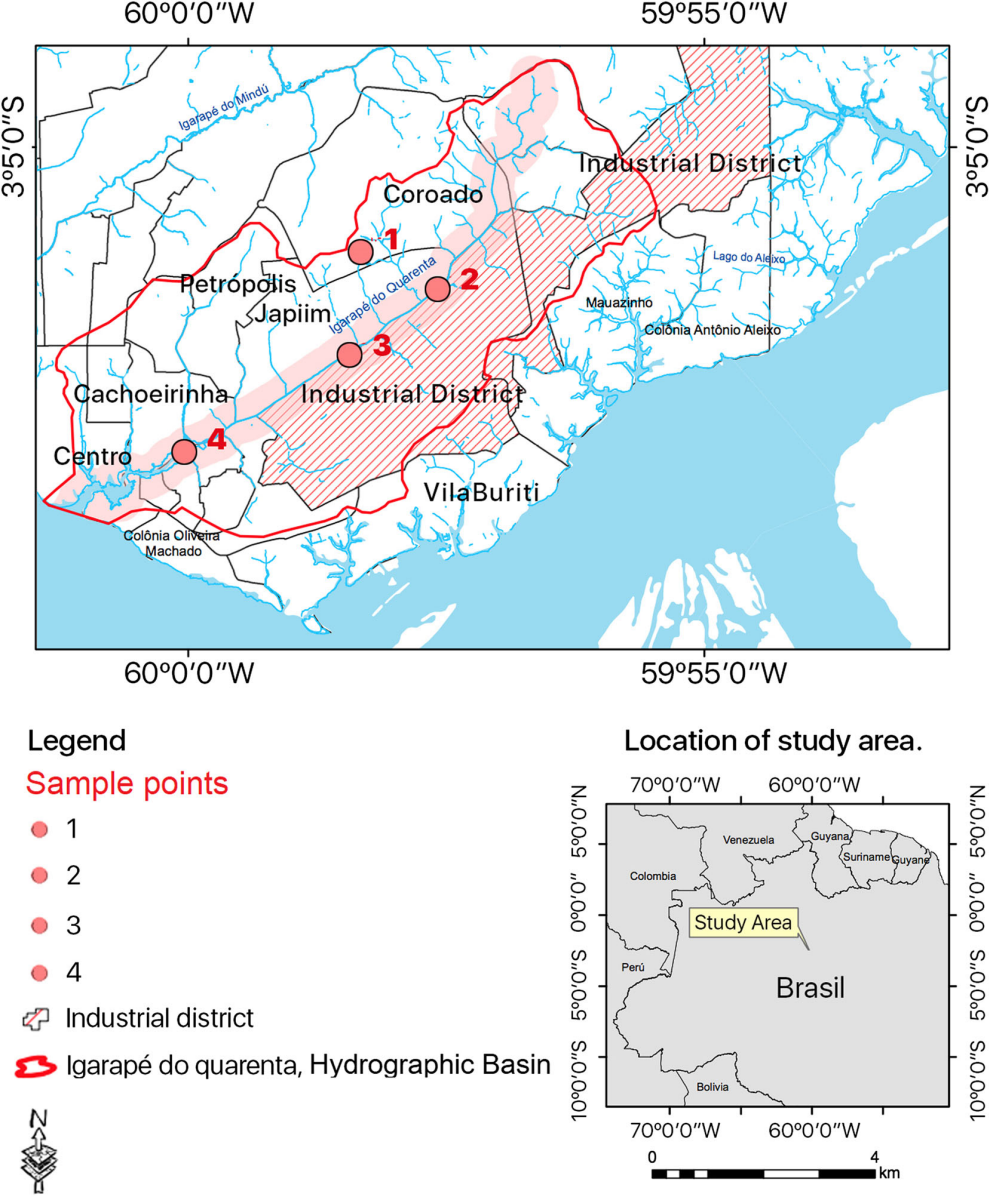
Igarapé do Quarenta (IgQ) hydrographic basin: area of study and sample collection. Molecular phylogenetic analysis using maximum likelihood method

Bacterial isolates were collected from water samples at four sites along the IgQ course in Manaus-AM (Fig. 1): sample 1—stream nascent at the Federal University of Amazonas, considered an unpolluted area (3° 06′ 00.44″ S, 59° 58′ 20.48″ O); sample 2—downstream (3° 06′ 25.57″ S, 59° 57′ 38.32″ O) at the Industrial District region, where industrial effluents and domestic sewage are discharged located on New Republic neighborhood (acronym NR at the samples codes); sample 3—downstream (3° 07′ 01.02″ S 59° 58′ 28.42″ O) area with increased discharge of domestic sewage and industrial effluents in the mediations of the State Department of Education and Quality of Teaching (SEDUC, acronym SC at the samples codes); and sample 4—down by (3° 07′ 56.14″ S, 60° 00′ 02.50″ O) area with huge discharge of domestic sewage nearby the debouch into the Igarapé do Educandos near a PROSAMIM (Social and Environmental Program of the Igarapés of Manaus, acronym AC at the samples codes) nucleus. Each site, with the aid of a Van Dorn bottle, was collected with 1 L of water. The water samples were collected according to the National Water Agency (ANA) recommendations (Brandão et al. 2011) and stored in refrigerated thermal boxes until their processing in laboratory.

### Bacterial Samples

Saline solution (0.9% NaCl) was used in the serial dilutions 1:10 at the ratios of (10^−1^, 10^−2^, 10^−3^, 10^−4^, and 10^−5^). For each dilution, 100-μL aliquot of the collected sample was plated, in triplicates, into Petri dishes containing tryptic soy agar (TSA), using the surface scattering technique, and the plates were incubated at 30 °C in a bacteriological incubator for 5 days. The microorganisms were categorized based on their morphology and staining characteristics using the Gram staining method. A small amount of each pure colony was transferred to 1.5-mL tubes containing TSA, medium supplemented with 20% glycerol, and cryopreserved at − 80 °C.

### Susceptibility to Hexavalent Chromium Cr(VI)

The collected colonies were standardized using the 0.5 McFarland turbidity standard and placed into sterile 96-well microplates for microdilution procedures. Potassium dichromate (K_2_Cr_2_O_7_) was used as the source of chromium(VI). The minimum inhibitory concentration (MIC) values were determined by observing the turbidity of the bacterial culture medium, which was plated on sterile 96-well microplates containing 195 μL of TSB broth supplemented with different concentrations of potassium dichromate (0.1, 1.0, 10, 15, 100, 200, 300, and 320 mg/L) added with 5 μL of each standardized isolate and incubated at 30 °C for 24 h (Giovanella et al. 2011; Netzahuatl-Muñoz et al. 2015; Upadhyay et al. 2017). Experiments were carried out in triplicate.

### Molecular Identification and Phylogeny

Genomic DNA of bacterial isolates resistant to Cr(VI) was extracted from fresh culture using the PureLink® Genomic DNA Mini Kit (Invitrogen by Thermo Fisher Scientific). The hypervariable regions from 16S rRNA gene were amplified by PCR with primers 400 F (5′-GAG AGT TTG ATC CTG GCT CAG-3′) and 1492 R (5′-CGG TGT GTA CAA GGC CCG GGA ACG-3′) using the Platinum® Taq DNA Polymerase kit (Invitrogen by Thermo Fisher Scientific) under the following conditions: 10 ng DNA template, 0.2 μM of each primer, 0.2 mM dNTPs, 1.5 mM MgCl_2_, 1X PCR buffer, 1 IU Taq DNA polymerase enzyme, and ultrapure water to the final volume of 25 μL. The cycling conditions were an initial denaturation at 95 °C for 3 min, followed by 30 cycles of 95 °C for 30 s, 59 °C for 30 s, 72 °C for 1 min, and a single 7-min step at 72 °C. PCR products were purified using the PureLink™ Quick PCR Purification kit (Invitrogen by Thermo Fisher Scientific) and sequenced with the BigDye Terminator v3.1 kit (Applied Biosystems™ by Thermo Fisher Scientific), following the manufacturer’s recommendations. Sequences were analyzed using the automated genetic analyzer ABI 3500 (Applied Biosystems™ by Thermo Fisher Scientific). The sequences were compared against the 16S ribosomal RNA sequences (bacteria and archaea) from NCBI—National Center for Biotechnology Information (http://ncbi.nlm.nih.gov)—using the BLASTn (Basic Local Alignment Search Tool).

The evolutionary history was inferred using the maximum likelihood method based on the Kimura 2-parameter model (Kimura 1980). This model showed the lowest BIC score (Bayesian Information Criterion) in the best model tool. The analysis involved 25 nucleotide reference sequences, chosen from BLASTn result for each bacterial isolate searching. The phylogeny was determined using the bootstrap method with 1000 replicates (Felsenstein 1985). Initial tree(s) for the heuristic search were obtained automatically by applying Neighbor-Join and BioNJ algorithms to a matrix of pairwise distances estimated by the maximum composite likelihood (MCL) method and then selecting the topology with superior log likelihood value. A discrete gamma distribution was used to model evolutionary rate differences among sites (5 categories (+G, parameter = 0.5573)). The rate variation model allowed for some sites to be evolutionarily invariable ([+I], 34.85% sites). All positions containing gaps and missing data were eliminated. Evolutionary analyses were conducted in MEGA7 (Kumar et al. 2016).

### Evaluation of Bacterial Isolates Potential to Degrade Cr(VI)

Cr(VI) quantification was performed using 3500-Cr D (diphenylcarbazide)—the standard colorimetric method for examination of water and wastewater (APHA 2012). All steps were followed to create the calibration curve (working range 0.01–1.0 mg/L). Percentage reduction was evaluated individually for each bacterial isolate after determination of MIC values. At the end of 72 h of culture, Cr(VI)-reduced concentration produced by each species was compared to control cultures (bacteria free).

#### Simulation of Cr(VI)-Contaminated Environment

In order to determine which one of the evaluated bacterial isolate was more resistant to chromium, a water environment contaminated with 10 and 300 mg/L Cr(VI) was simulated.

Culture media were prepared with water collected in the IgQ nascent. The volume of water collected was filtered in 1.2-μm pre-filter for removal of macroparticulates, and then in 0.45-μm filter for removal of suspended particulate material, remaining only water-dissolved components as ions, metals, and organic compounds. Then, the filtrated water sample was autoclaved for 15 min at 121 °C.

The inoculum density was standardized in spectrophotometer by measuring absorbance at 600 nm. A first experiment was performed to evaluate chromium degradation in medium similar to that Cr(VI)-contaminated water environment, with or without addition of nutrients (Luria-Bertani medium—LB). The experiments were performed, in duplicate, using sterile beakers and cups oxygenated by compressors containing a 250-mL sample. Nascent water added with 10 mg/L Cr(VI) was used as control and for the experiments nascent water + 10 mg/L Cr(VI) + LB medium + bacterial isolate and nascent water + 10 mg/L Cr(VI) + bacterial isolate.

A second experiment was carried out, in quadruplicate, to compare the growth of the selected isolates cultured in medium free of chromium and in medium added with 300 mg/L Cr(VI). Samples were incubated in a rotary shaker (Shaker-CE-725) at 30 °C and 130 rpm. Growth curves based on the culture absorbance ratio were determined using a wavelength spectrophotometer at 600 nm, in 24-h intervals.

Data of Cr(VI) concentrations from the two experiments, using the diphenylcarbazide method, as well as the pH measurement in experiments using 10 mg/L Cr(VI), were obtained every 12 h up to the final experimental period (72 h). The first measure occurred prior to the addition of the selected bacterial isolate. Shapiro-Wilk test was performed to evaluate normality of each replicate and the OriginPro 8 software was used for data analysis and graph plotting.

## Results and Discussion

### Susceptibility of Microorganisms to Cr(VI)

Eighty-four microorganisms were isolated from the IgQ and all of them showed resistance to Cr(VI) at concentrations of 0.1, 1.0, 10, 15, and 100 mg/L of K_2_Cr_2_O_7_. From those 84 isolates, 35 exhibited resistance at concentrations up to 200 mg/L and only 23 isolates resisted to the concentration of 300 mg/L (~ 106 mg/L Cr^6+^).

### Molecular Identification and Phylogeny of Isolates Resistant to Cr(VI)

**Fig. 2:**
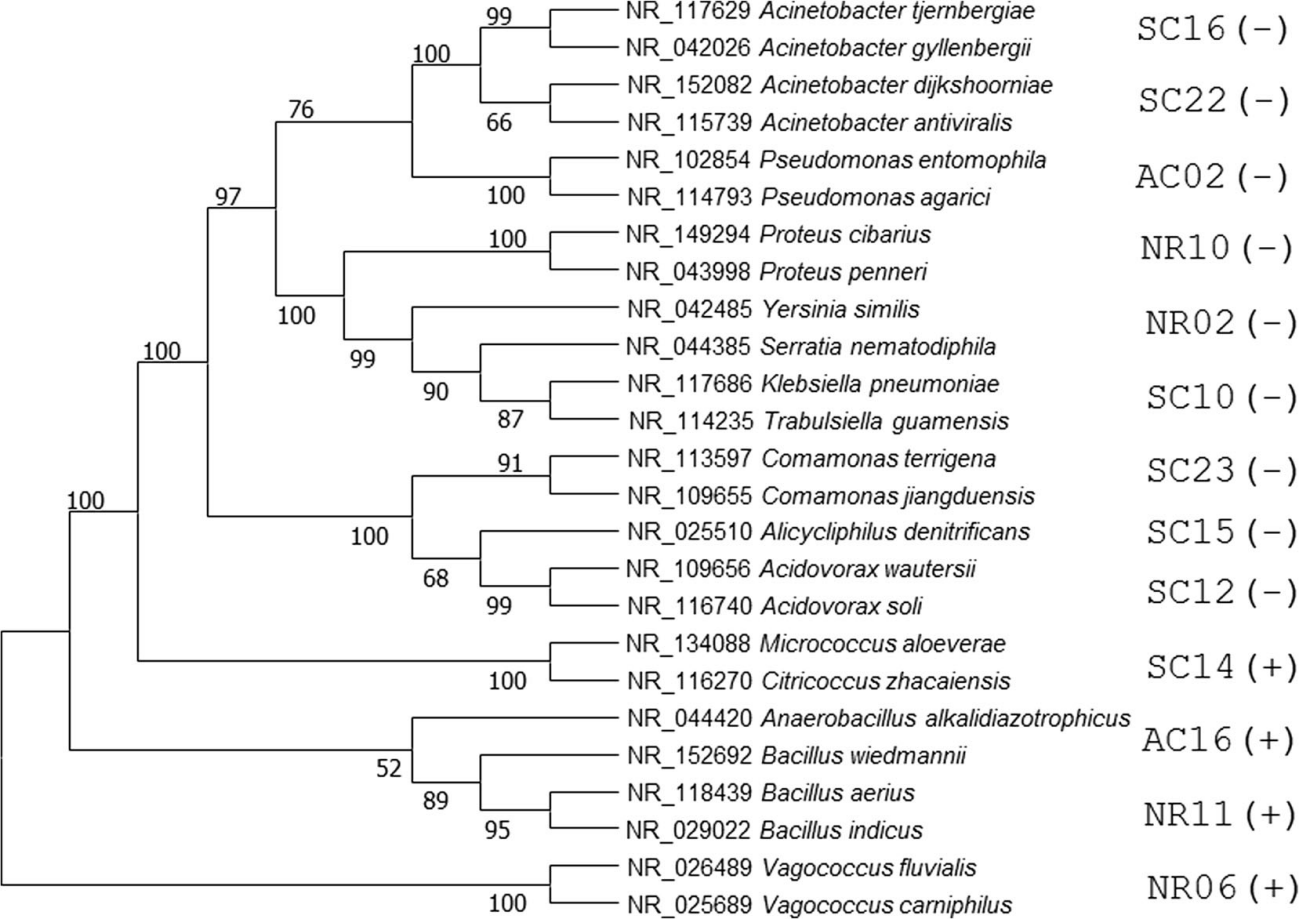
Molecular phylogenetic analysis using maximum likelihood method. Gram stain result: (+) and (−)

Seventeen isolates, which exhibited greater chromium resistance, were grouped in 13 genera. Despite the limitation in identifying the isolates at the species level, the phylogenetic analysis showed consistent and distinct pattern for bacteria diversity (Fig. 2), most of them within the phylum Proteobacteria of the class Gammaproteobacteria. The details of the identification of the isolates resistant to Cr(VI) are described in Supplementary Table 1. Only one isolate for each cluster is shown on the phylogenetic tree, i.e., isolates with 100% 16S rRNA gene sequence identity, specifically SC10/AC8 and NR10/NR13/NR16/NR19. All other isolates were not clustered using this criterion and therefore their sequences were clustered separately for the construction of phylogenetic trees. Using Gram staining technique, it was possible to determine that among isolates of 10 genera, 6 were Gram-negative bacillus, 2 Gram-positive cocci, 1 Gram-positive bacillus, and only 1 Gram-negative coccobacillus.

Obtained results corroborate Bizani and Spagiari (2016) findings, who reported the resistance of *Acetobacter aceti* and *Micrococcus luteus* to hexavalent chromium at the concentration of 300 mg/L and metal reduction capacity of 36 and 38%, respectively. Also, Upadhyay et al. (2017), investigating *B. subtilis* MNU16 tolerance to Cr(VI) and its reduction potential in concentrations of 50–300 mg/L, observed that the isolate tolerance limit was 300 mg/L Cr(VI). Moreover, Batool et al. (2014) evaluated the resistance of *Pseudomonas aeruginosa* and *Ochrobactrum intermedium* strains to hexavalent chromium at concentrations of 100, 500, and 1000 μg/mL and concluded that Cr(VI) stress significantly influence the morphology of bacteria altering characteristics of shape and size. The reported ability of some specific bacteria to tolerate high concentrations of Cr(VI) evidences their potential to bioremediate chromium-contaminated environments.

### Ability of IgQ Isolates to Reduce Cr(VI) at the Concentration of 300 mg/L

**Fig. 3:**
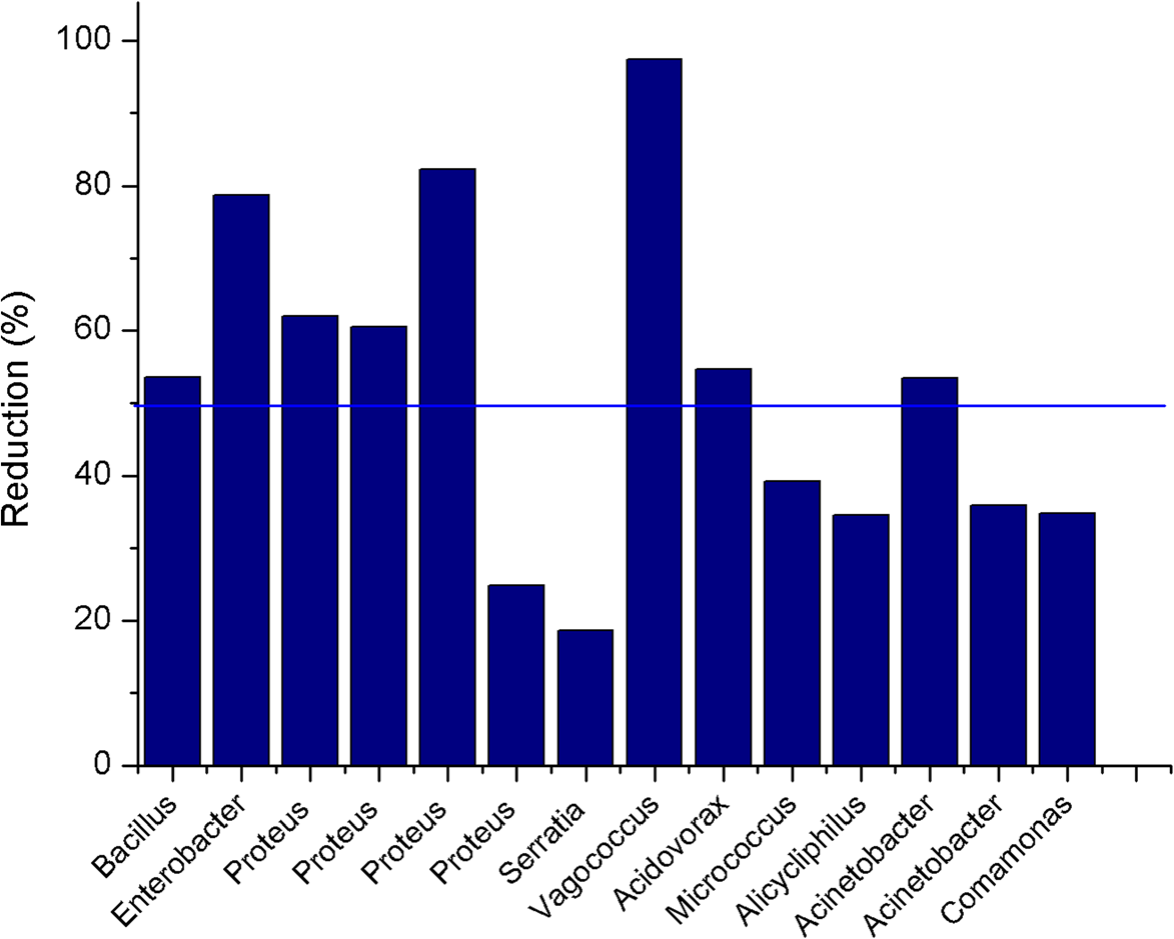
Rates of Cr(VI) after 72 h cultivation with IgQ isolates

The efficiency of IgQ isolates in reducing Cr(VI) determined by s-diphenylcarbazide colorimetric method is shown in Fig. 3, which exhibits the profile of the isolates more resistant to hexavalent chromium after an exposure period of 72 h. It was evidenced that the isolates of the genera *Vagococcus* sp. (NR06), *Proteus* sp. (NR16), *Enterobacter* sp. (AC8), and *Bacillus* sp. (NR10) collected at the IgQ sites 2 and 4 and cultured in TSB culture medium at 30 °C, reduced 97.4, 82.3, 78.7%, and 53.6% of Cr(VI) content, respectively, and were therefore considered the most effective.

Other genera such as *Bacillus* sp., *Pseudomonas* sp., *Escherichia coli*, and *Proteus* sp. were also investigated at similar concentrations and were efficient in bioremediation processes of wastewaters contaminated with Cr(VI) (Upadhyay et al. 2017; Drzewiecka 2016).

*Serratia* sp. and *Proteus* sp., both collected at IgQ site 2, showed lower efficiency in reducing chromium levels, 18.6 and 24.8%, respectively. It is probable that though their tolerance to heavy metals is high, they are not efficient to degrade chromium. Hacioglu and Tosunoglu (2014) also reported that the structure and function of microbial communities may be affected by heavy metals, resulting in mutations that influence adaptability or cause the microorganisms’ death in heavy metal-impacted environments.

Isolates of the genera *Acidovorax* sp., *Micrococcus* sp., *Acinetobacter* sp., *Alicycliphilus* sp., and *Comamonas* sp., frequently found at IgQ site 3, exhibited Cr(VI)-degrading ability in the range 34.5–54.7%. Studies carried out by Dimitroula et al. (2015), Somenahally et al. (2013), Puyen et al. (2012), Srivastava and Thakur (2007), Liu et al. (2015), and Bestawy et al. (2013) investigated the role of microorganisms of those genera in Cr degradation processes. This work reports a first study on the potential of isolates of different genera collected in Amazon, which can be used in bioremediation processes.

### Growth Profile of *Vagococcus* sp.

**Fig. 4:**
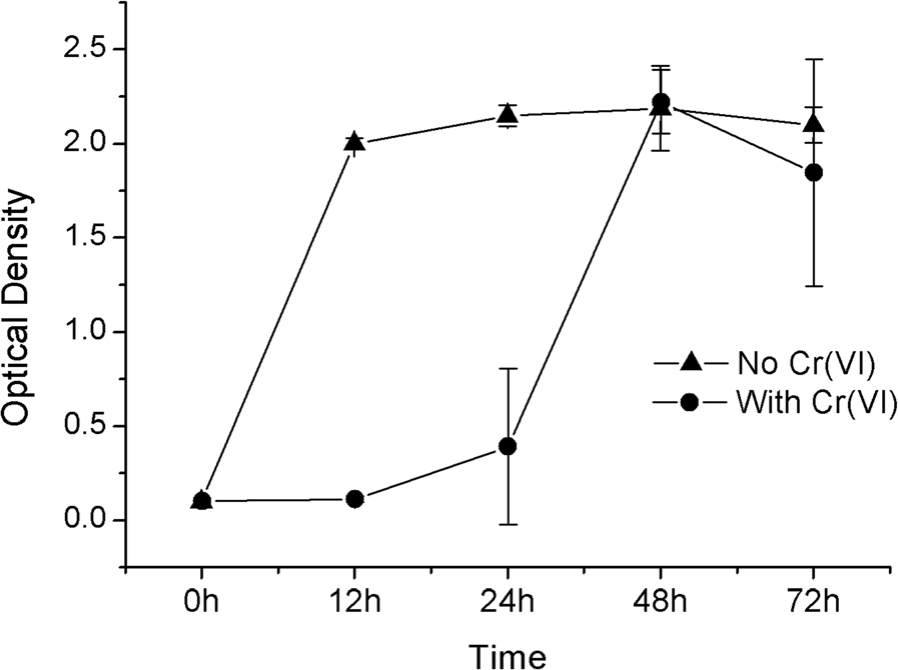
Growth rates of *Vagococcus* sp. isolates in absence of chromium(VI) (-▲-) and in the presence of chromium(VI) (-●-) during 72 h with optical density measurement

The genus *Vagococcus* is composed by Gram-positive bacteria formed by non-spore non-mobile cocci (Wang et al. 2011). According to Coleman (1988), Gram-positive bacteria are more resistant to high concentrations of Cr(VI) if compared to Gram-negative bacteria. *Vagococcus* sp. cultured in medium devoid of chromium, in the first 12 h, showed enhanced growth compared to that exhibited for isolates cultured in the presence of Cr(VI) (Fig. 4).

When exposed to Cr(VI), *Vagococcus* sp. isolates underwent an adaptation process during the first 24 h. Only after that period of metabolic adjustment was evidenced their ability to degrade chromium. The maximum growth and adaptation of *Vagococcus* sp. to the chromium-supplemented medium were reached in 48 h, evidencing its potential for bioremediation of effluents contaminated with Cr(VI). Shakoori and Muneer (2002) reported that *Vagococcus* sp. was able to degrade Cr(VI) to its nontoxic trivalent form. Those results reinforce the potential use of bacterial isolates on the decontamination of polluted environments and that bioremediation can be a strategic waste management tool harmless to animals, plants, and humans.

### Assay Carried out with *Vagococcus* sp. in an IgQ-Simulated Environment

**Fig. 5:**
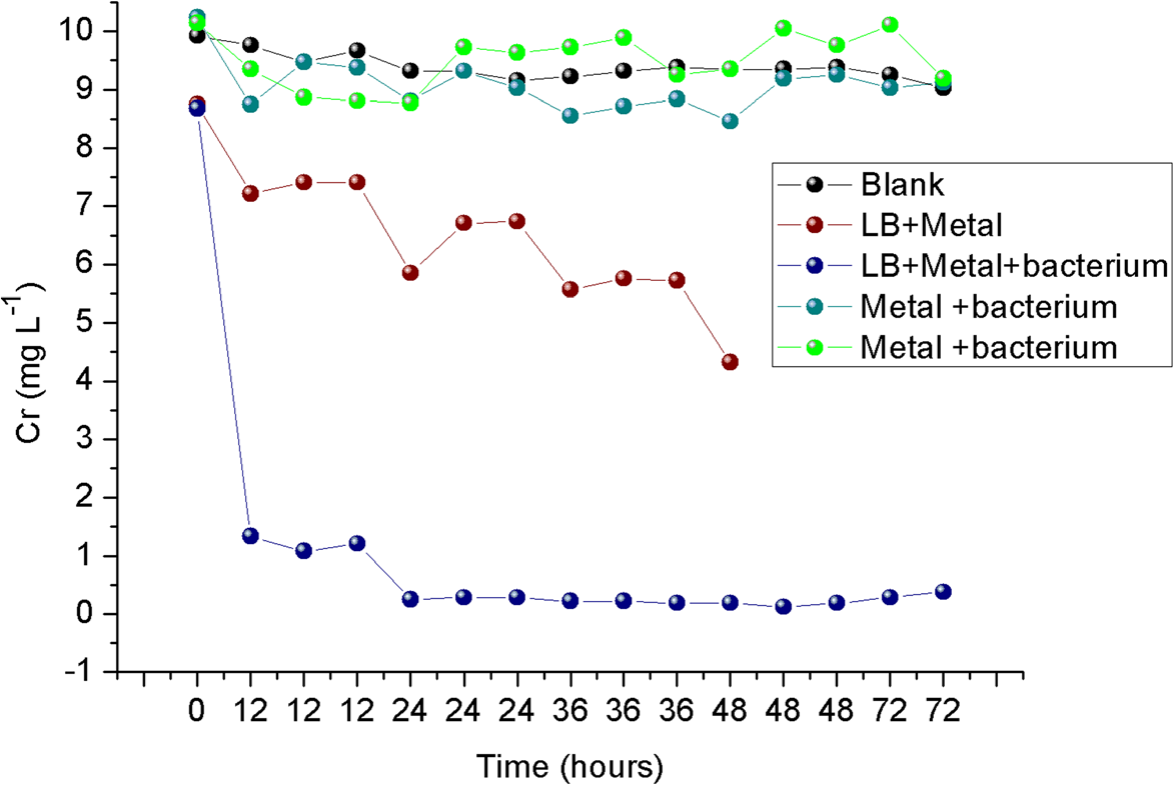
Changes on Cr(VI) concentration during 72 h of culture of *Vagococcus* sp. isolates in medium simulating environmental conditions and in LB culture medium

No Cr(VI) contamination was observed in IgQ waters when collecting the biological samples, but historical monitoring records of that bayou waters presented high levels of total chromium (between 158.4 ± 164.6 mg/L), according to Pio et al. (2013). The National Environmental Council legislation in force in Brazil (CONAMA 2011), for effluent releases, establishes that the maximum level of Cr(VI) permitted is 0.1 mg/L. That legally recognized rate increased 100 times could simulate a contaminated environment. Therefore, to investigate the degrading potential of *Vagococcus* sp. isolates, two distinct conditions were established: IgQ water added with 10 mg/L Cr(VI) and LB culture medium supplemented with IgQ and 10 mg/L Cr(VI). *Vagococcus* sp. isolates cultured in LB medium practically reduced total chromium after 12 h of incubation while the isolates cultured in IgQ water degraded only small quantity of the metal (Fig. 5).

Experiments were carried out for 72 h. Normality was determined by Shapiro-Wilk test (*p* < 0.05), replicates (*N* = 15), and a normal distribution with *W* values (0.9248 and 0.9297) higher than critical value (0.881), indicated the normality of the sample set. Variance analysis showed that in the absence of nutrients, the populations were not significantly different (*F* statistics = 0.9029, *p* value = 0.851) and presented an average of 10.14% Cr reduction. Conversely, addition of nutrients in the medium promoted 96.18% Cr(VI) degradation after 72-h cultivation and 86.11% after the first 12 h. To better adapt to chromium-impacted environments (simulated or real), bacteria requires a medium containing basic nutrients such as those found in unadulterated environments, so that their basic physiological needs can be maintained such as pH, temperature, bioavailability of minerals, and nutrients (Olszewska et al. 2016).

Obtained results indicated that the nutrient medium devoid of bacterial inoculum also reduced Cr(VI). Experiments using only the LB basic medium after 24 h of culture presented a Cr(VI) reduction of 23%. However, the highest chromium degradation potential was obtained in cultures added with *Vagococcus* (97.4%). Pal and Paul (2004) reported that the addition of nutritional supplements to the medium increases degradation efficiency and they demonstrated that glucose (1.0 g/L) added to the culture medium enhanced chromium degradation up to 55.5% after 24 h of incubation.

**Fig. 6:**
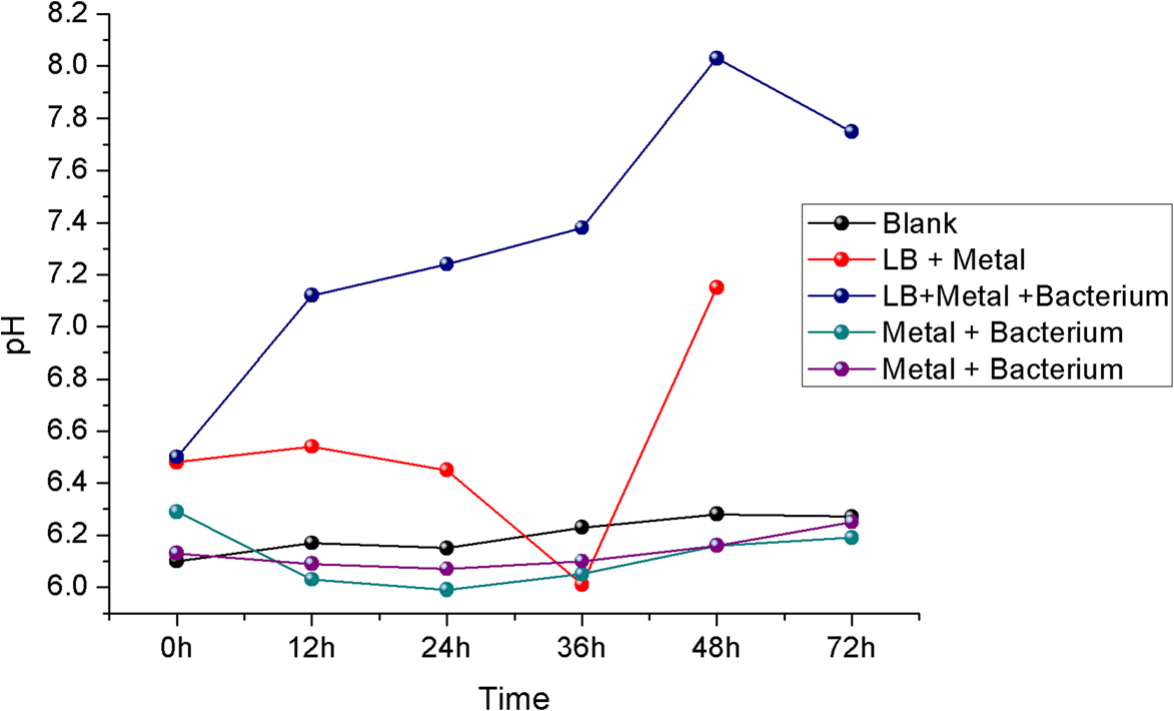
pH changes in the different simulated conditions

Regarding pH values, the *Vagococcus* sp. isolates cultured in LB medium added with chromium reacted differently compared to other strains (Fig. 6). The pH of the medium altered from 6.5 to 7.75 at the end of the experiment. Brandhuber et al. (2004) reported that this pH change may be explained by the Cr(VI) reduction reaction that occurs as follows: CrO_4_^2−^ + 8H^+^ + 3e^−^ → Cr^3 +^ + 4H_2_O.

According to Conceição et al. (2007) and Somenahally et al. (2013), microorganisms have the ability to adapt to various environmental changes, as long as they are able to maintain the intracellular pH around 7.5, a process that is achieved by the extrusion or intrusion of H^+^ ions by the microbial cell. A trend curve following the equation *y* = − 0.08235*x* + 8.4706 and *R*^2^ = 0.93658 was established to validate pH values obtained in the experiment and it allowed to estimate that the concentration of Cr(VI) would reach zero after 102 h. However, degradation of metal in culture of *Vagococcus* sp. isolates in medium containing chromium occurred within a maximum of 24 h.

## Conclusion

Obtained results indicated that *Enterobacter* sp., *Bacillus* sp., *Proteus* sp., *Acidovorax* sp., *Acinetobacter* sp., and *Vagococcus* sp. isolates collected in the IgQ are effective on the bioremediation of environments and effluents contaminated with Cr(VI). Moreover, *Vagococcus* sp. isolates showed significant ability to degrade (Cr) or Cr(VI) at an initial concentration of 10 mg/L (96.8% within 24 h) and at higher concentration (300 mg/L); after 72 h, those isolates drastically reduced (97.4%) the metal. Those findings indicate that additional investigations on the pathways for chromium removal by *Vagococcus* sp. should be carried out. Furthermore, the efficiency of a consortium of isolates of different strains should be considered to investigate its ability to bioremediate Amazonian environments contaminated with heavy metals.

## Electronic Supplementary Material

Supplementary Table 1 (PDF 70 kb)
