# Supplementary material for: Potential of Bacterial Isolates from a Stream in Manaus-Amazon to Bioremediate Chromium-Contaminated Environments
Source: Water Air Soil Pollut. 2018 Jul 31;229(8):266. doi: 10.1007/s11270-018-3903-1 (PMC6096555; doi:10.1007/s11270-018-3903-1)
Supplement: Supplementary file 1 — (PDF 70 kb) [file 11270_2018_3903_MOESM1_ESM.pdf]

Table 1: Characterization by similarity obtained from 16S rDNA gene sequences comparison from bacteria isolated from sections of the IgQ with the contained GenBank database sequences through the BLASTn tool.

Code	Strain	Collection Site	Similar Strain	Accession number	Identity (%)
NR02	02	Nova República	*Serratia* sp.	NR_044385	100
NR06	06	Nova República	*Vagococcus* sp.	NR_026489	100
				NR_025689	99
NR10	10	Nova República	*Proteus sp.*	NR_149294	100
NR11	11	Nova República	*Bacillus sp.*	NR_118439	100
NR13	13	Nova República	*Proteus sp.*	NR_149294	100
NR16	16	Nova República	*Proteus sp.*	NR_149294	100
NR19	19	Nova República	*Proteus sp.*	NR_149294	100
SC10	10	Seduc	*Klebsiella sp.*	NR_117686	100
SC12	12	Seduc	*Acidovorax* sp.	NR_109656	99
				NR_116740	
SC14	14	Seduc	*Micrococcus* sp.	NR_134088	100
SC15	15	Seduc	Alicycliphilus sp.	NR_025510	100
SC16	16	Seduc	*Acinetobacter* sp.	NR_117629	100
				NR_042026	
SC22	22	Seduc	*Acinetobacter* sp	NR_152082	99
SC23	23	Seduc	*Comamonas* sp.	NR_113597	99
				NR_109655	
AC02	02	Prosamim	*Pseudomonas* sp.	NR_102854	99
				NR_114793	98
AC08	08	Prosamim	*Enterobacter* sp.	NR_146667	99
AC16	16	Prosamim	*Bacillus* sp.	NR_152692	99
